# Ocular Motor Cranial Nerve Palsies and Increased Risk of Primary Malignant Brain Tumors: South Korean National Health Insurance Data

**DOI:** 10.3390/cancers16040781

**Published:** 2024-02-14

**Authors:** In Jeong Lyu, Kyungdo Han, Kyung-Ah Park, Sei Yeul Oh

**Affiliations:** 1 Department of Ophthalmology, Korea Cancer Center Hospital, Seoul 01812, Republic of Korea; ijlyu@kirams.re.kr; 2 Department of Statistics and Actuarial Science, Soongsil University, Seoul 06978, Republic of Korea; hkd@ssu.ac.kr; 3 Samsung Medical Center, Sungkyunkwan University School of Medicine, Seoul 06351, Republic of Korea

**Keywords:** cranial nerve palsy, ocular motor cranial nerve palsy, brain tumor, central nerve system tumor, health insurance data

## Abstract

**Simple Summary:**

Established risk factors for primary malignant brain tumors include hereditary syndromes, family history of brain tumors, head trauma, smoking, alcohol consumption, radiation exposure, electromagnetic wave exposure, infections, inflammation, and exposure to toxic substances. However, the impact of acquired ocular motor cranial nerve palsies on subsequent primary malignant brain tumors remains underexplored. Our research presents an exhaustive analysis of data from the Korea National Health Insurance Service from 2010 to 2017. Correlation between ocular motor cranial nerve palsies and emergence of primary malignant brain tumors within a Korean population was investigated. Our findings emphasize an amplified risk of primary malignant brain tumors in patients diagnosed with ocular motor cranial nerve palsies, particularly among women aged below 65.

**Abstract:**

The aim of this study was to investigate the association between ocular motor cranial nerve palsies (OMCNP) and the occurrence of primary malignant brain tumors in a Korean population, using the national sample cohort database from Korea National Health Insurance Service (KNHIS). KNHIS data between 2010 and 2017 were analyzed. Our sample encompassed 118,686 participants, including 19,781 from a recently diagnosed OMCNP cohort and 98,905 from a matched control cohort through a 1:5 propensity score matching based on age and gender. To counteract the issue of reverse causation, we integrated a one-year time lag in our sensitivity analysis. Study participants were followed up until 31 December 2019. Cox proportional hazard regression analysis was used to compute the adjusted hazard ratio (HR) for primary malignant brain tumors according to the OMCNP diagnosis. Additionally, we performed a subgroup analysis to discern effects of various factors on the association between OMCNP and primary malignant brain tumors. HR for primary malignant brain tumors was 3.272 (95% confidence interval [CI]: 2.294 to 4.665) in the OMCNP cohort compared to the control cohort in a fully adjusted model for age, sex, socio-economic status, smoking, drinking, regular physical exercise, hypertension, diabetes, dyslipidemia, obesity, chronic kidney disease, and human immunodeficiency virus infection. Further subgroup analysis revealed that the risk of primary malignant brain tumors was significantly increased in women with OMCNP compared to men with OMCNP (HR: 5.118 in women vs. 2.441 in men, *p* = 0.0440), and in those aged <65 years than in those aged ≥65 years (HR: 6.951 in age < 65 years vs. 1.899 in age ≥ 65 years, *p* = 0.0006). Our population-based cohort study demonstrated a significantly increased risk of subsequent primary malignant brain tumors in patients with OMCNP. Particularly, OMCNP-afflicted women aged below 65 manifested a heightened probability of developing primary malignant brain tumors compared to those devoid of OMCNP.

## 1. Introduction

Malignant brain tumors are fatal and crucial diseases that can be accompanied by serious neurological complications [1,2,3,4]. Given their critical nature, timely monitoring and early detection are essential for high-risk individuals. Although malignant brain tumors are composed of over 100 diverse histologically distinct subtypes [5,6], established risk factors for primary malignant brain tumors include hereditary syndromes (such as tuberous sclerosis and neurofibromatosis); family history of brain tumors; head trauma; smoking; alcohol consumption; radiation exposure; electromagnetic wave exposure; infections; inflammation; and exposure to toxic substances [7,8,9,10,11,12,13].

The ocular motor cranial nerve is composed of the third (oculomotor), fourth (trochlear), and sixth (abducens) cranial nerves. The third nerve arises from the interpeduncular fossa of the midbrain, passes along the lateral wall of the cavernous sinus and through the superior orbital fissure, and innervates the levator palpebrae superioris and most extraocular muscles including the superior, medial, inferior rectus, and inferior oblique muscles. It also has parasympathetic functions, controlling ciliary and sphincter pupillae muscles. The fourth nerve arises from the dorsal surface of the midbrain just below the inferior colliculus, surrounds the contralateral surface of the midbrain, passes along the lateral wall of the cavernous sinus and through the superior orbital fissure, and innervates the superior oblique muscles. Lastly, the sixth nerve arises from the pontomedullary junction near the midline, crosses the petrous apex within Dorello’s canal, ascends to pass through the cavernous sinus near internal carotid arteries, exits through the superior orbital fissure, and innervates the lateral rectus muscle [14].

Diplopia is disturbing and the most common symptom of acquired ocular motor cranial nerve palsies (OMCNP). Ptosis or mydriasis can be accompanied by third cranial nerve palsy [15,16,17,18]. The etiology of OMCNP includes microvascular ischemia, aneurysm, stroke, inflammation, infection, and trauma. Brain tumors are also well-known causes of OMCNP [15,16,17,18,19]. However, reversely, the impact of OMCNP on subsequent primary malignant brain tumors remains underexplored.

Therefore, this study aimed to ascertain the effect of OMCNP on the incidence of primary malignant brain tumors using the National Sample Cohort (NSC) database from Korea’s National Health Insurance Service (KNHIS).

## 2. Materials and Methods

### 2.1. Data Source

This study used nationwide population data from the Korean National Health Insurance Service (KNHIS) claims database. KNHIS covers approximately 97% of Korea’s population of 50 million and gathers all health care utilization information for inpatients and outpatients using codes from the Korean Standard Classification of Diseases (KCD), 7th Revision, based on the International Classification of Diseases, 10th Revision. KNHIS also covers a biennial national health screening program including a self-questionnaire on health behavior, past medical history, general body measurements, and laboratory tests for all beneficiaries aged ≥20 years.

We used a customized KNHIS database cohort that included 40% of the Korean population selected via stratified random sampling to ensure that the sample represented the Korean population well. We reviewed the data of both inpatient and outpatient claims, including demographics, socioeconomic status, laboratory test findings, diagnosis of disease, medical treatment, and procedure.

### 2.2. Study Population

This study included individuals aged ≥20 years with newly diagnosed OMCNP based on the ICD-10-CM codes between 2010 and 2017, who underwent a national health screening examination within 2 years prior to the diagnosis of OMCNP. Those with ICD-10-CM codes of H49.0 (third cranial nerve palsy), H49.1 (fourth cranial nerve palsy), and H49.2 (sixth cranial nerve palsy) were enrolled. We excluded anyone with myasthenia gravis (G70.0), dysthyroid exophthalmos (H06.2), or thyrotoxicosis (E05). Those with a history of any type of cancer at any time before enrollment were also excluded. The control cohort was selected by a 1:5 propensity score matching based on gender and age of individuals not diagnosed with OMCNP (19,781 individuals with OMCNP and 98,905 controls). The primary end point was the incidence of newly diagnosed primary malignant brain tumors. Primary malignant brain tumor was defined as a malignant neoplasm of the meninges (C70), brain (C71), spine or other central nervous systems (C72), along with a special claim for cancer (V193). We excluded benign brain tumors due to different clinical behavior and growth patterns, including mortality and morbidity between malignant and nonmalignant tumors [4,13,20]. Metastatic brain tumors were also excluded because of their different etiologies and histological variance depending on the origin of the primary tumor.

We used a 1-year time lag in sensitivity analysis to avoid the potential problem of reverse causation. Study participants were followed up until 31 December 2019.

This study was approved by the Samsung Medical Center (SMC) Institutional Review Board (IRB) (IRB no. SMC 2022-12-021). It was performed in accordance with the principles of the Declaration of Helsinki. The requirement for informed consent from individual patients was waived because the data used were public and anonymized under confidentiality guidelines.

### 2.3. Variables

When systolic blood pressure was 140 mmHg or more, diastolic blood pressure was 90 mmHg or more, or there was at least one claim per year for an antihypertensive medication prescription under ICD-10-CM codes I10-I13 or I15, hypertension was considered. When a fasting glucose level was 126 mg/dL or more, or there was at least once claim per year for a prescription of hypoglycemic agents under ICD-10-CM codes E11 to E14, diabetes mellitus (DM) was considered. When total cholesterol level was 240 mg/dL or more, or there was at least one claim per year for an antihyperlipidemic medication prescription under ICD-10 code E78, dyslipidemia was considered. When an eGFR level was less than 60 mL/min/1.73 m^2^, chronic kidney disease was considered. If a claim had an ICD-10-CM code B20 (HIV disease resulting in infectious and parasitic diseases), human immunodeficiency virus (HIV) infection was considered. Height and weight were measured using an electronic scale at a medical facility during a health examination. Body mass index (BMI) was calculated as body weight (kg) divided by height squared (m^2^). Based on World Health Organization recommendations for Asian populations, obesity was considered when BMI was 25 kg/m^2^ or more. Low income was defined as having income at the bottom 20% level of the total population.

A standardized self-reported questionnaire was used to evaluate general health behaviors and lifestyles. Smoking status was categorized into smokers and nonsmokers/former smokers. Drinking status was classified as drinkers and nondrinkers. Regular physical activity was defined as doing high-intensity exercise for at least 20 min a day, three times a week or 30 min of moderate-intensity exercise per day, five times a week.

### 2.4. Statistical Analysis

Baseline characteristics of the OMCNP cohort and control cohort were compared using the chi-squared test for categorical variables and analysis of variance (ANOVA) for continuous variables. Data were presented either as numbers with their associated percentages or as a mean with standard deviations. Incidence rates for primary malignant brain tumors were determined by dividing the number of events by 1000 person-years. Multivariable Cox proportional hazards regression analysis was performed to evaluate the association between OMCNP and the risk of primary malignant brain tumors with the hazard ratio (HR) and 95% confidence interval (CI) calculated. To adjust for potential confounders, three models were established. Model 1 was unadjusted. Model 2 was adjusted for age and sex. Model 3 was fully adjusted for age, sex, low income, smoking, drinking, regular physical exercise, hypertension, diabetes, dyslipidemia, obesity, chronic kidney disease (CKD), and HIV infection. All statistical analyses were carried out using SAS software version 9.4 (SAS Institute Inc., Cary, NC, USA).

## 3. Results

**Figure 1:**
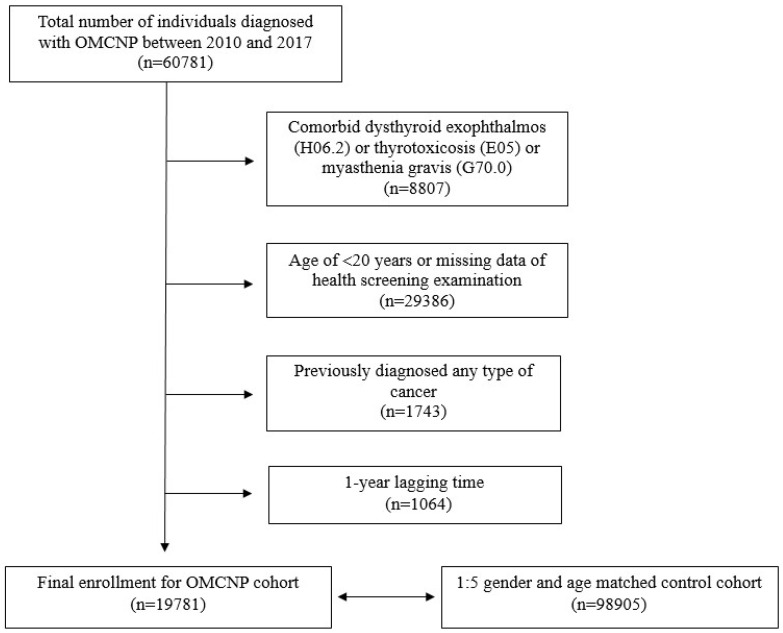
Flow chart showing enrollment of current study cohort.

Between 2010 and 2017, 60,781 individuals received a new OMCNP diagnosis. After applying exclusion criteria and selecting a control cohort through a 1:5 propensity score matching based on age and gender, a total of 118,686 participants were enrolled in this study, including 19,781 from the OMCNP cohort and 98,905 controls (Figure 1). Demographic characteristics of participants with OMCNP and the control cohort are presented in Table 1.

There were no significant differences in age, sex, smoking status, regular physical activity, or HIV infection between the two groups. However, comorbidities such as diabetes, hypertension, dyslipidemia, chronic kidney disease, and obesity were more common in the OMCNP cohort. On the other hand, drinking and lower income rates were higher in the control cohort.

### 3.1. Risk of Primary Malignant Brain Tumors According to the Presence of OMCNP

Table 2 displays the incidence rate and HRs for the development of primary malignant brain tumors. The Cox proportional hazards model revealed that the HR for primary malignant brain tumors in the OMCNP cohort was significantly higher in all models. In model 1 without adjustment, patients with OMCNP had an HR of 3.239 (95% CI: 2.287–4.589) for primary malignant brain tumors compared to the control cohort. After adjusting for age and sex (model 2), HR was 3.241 (95% CI: 2.288–4.591). In a fully adjusted model (model 3, adjusted for age, sex, low-income, smoking, drinking, regular physical exercise, hypertension, diabetes, dyslipidemia, obesity, CKD, and HIV infection), the HR was 3.272 (95% CI: 2.294–4.665).

**Figure 2:**
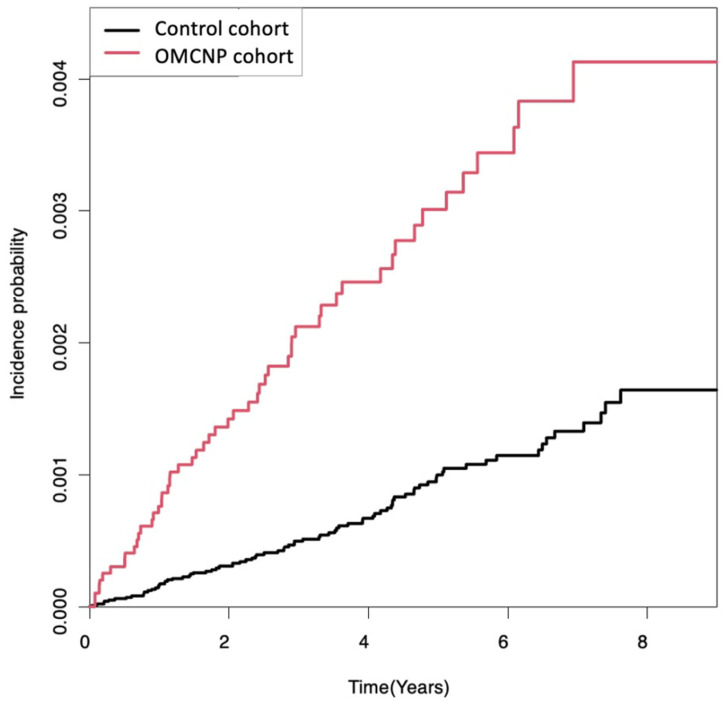
Kaplan-Meier survival curves of the incidence probability of primary malignant brain tumors for up to 9 years in participants with cranial nerve palsies and controls. There was an increased risk of developing primary malignant brain tumors during the follow-up period in the OMCNP cohort compared to the control cohort (log-rank *p* < 0.0001). OMCNP, ocular motor cranial nerve palsies.

Cumulative incidence of primary malignant brain tumors in the OMCNP cohort and control cohort are shown in Figure 2. Primary malignant brain tumors occurred more frequently in the OMCNP cohort than in the control cohort during a follow-up period up to 9 years (log-rank *p* < 0.0001).

### 3.2. Hazard Ratio with a Further Time Lag

When a 3-year time lag was applied, the HR for primary malignant brain tumors in the OMCNP cohort was 2.425 (95% CI: 1.442–4.078) in model 1, and 2.425 (95% CI: 1.442–4.078) in model 2, compared to the control cohort. In the fully adjusted model, HR was 2.39 (95% CI: 1.406–4.061). When a 5-year time lag was applied, the HR was 2.041 (95% CI: 0.899–4.634) in model 1, 2.042 (95% CI: 0.900–4.637) in model 2, and 2.055 in the fully adjusted model (95% CI: 0.892–4.735) (Table 3).

### 3.3. Locations of Primary Malignant Brain Tumors

Locations of primary malignant brain tumors in participants with OMCNP and controls are presented in Table 4. The OMCNP cohort showed a higher prevalence of primary malignant tumors in the meninges compared to the control cohort, although the brain parenchyma was the most common site in both groups (*p* = 0.0047).

### 3.4. Subgroup Analysis

Subgroup analysis was performed to evaluate the effects of other factors (including age, sex, smoking status, drinking status, regular physical activities, low income, and other comorbidities) on the association of OMCNP with primary malignant brain tumors. Younger than 65 years (HR: 6.951, 95% CI: 3.995–12.094 in age <65 years vs. HR: 1.899, 95% CI: 1.158–3.114 in age ≥65 years, *p* = 0.0006) and women (HR: 5.118, 95% CI: 2.933–8.932 in women vs. HR: 2.441, 95% CI: 1.532–3.889 in men, *p* = 0.0440) were significant interaction factors between OMCNP and primary malignant brain tumors (Table 5).

## 4. Discussion

Results of this study showed an increased risk of primary malignant brain tumors within the OMCNP cohort during a follow-up period of up to 9 years. Compared to the control cohort, the OMCNP cohort demonstrated a 3.372-fold increase in the risk of primary malignant brain tumors, even after adjusting for variables such as age, sex, socio-economic status, smoking, drinking, regular physical exercise, and other comorbidities (obesity, diabetes, hypertension, dyslipidemia, chronic kidney diseases, and HIV infection). In particular, the OMCNP cohort displayed a marked surge in primary malignant brain tumor risk among women compared to men (HR: 5.118 in women vs. 2.441 in men, *p* = 0.0440) and those aged below 65 years versus those aged 65 years or more (HR: 6.951 in age < 65 years vs. 1.899 in age ≥ 65 years, *p* = 0.0006). We adopted a 1-year lag post OMCNP diagnosis before the emergence of primary malignant brain tumors to exclude reverse causation, in order to analyze the effect of OMCNP on primary malignant brain tumors. In addition, further temporal analyses incorporating 3-year and 5-year lag periods were conducted to sharpen our understanding of OMCNP’s potential role in brain tumor genesis. The risk of developing a brain tumor after OMCNP decreased slightly from 3.27 with a 1-year lag to 2.39 with a 3-year lag and 2.06 with a 5-year lag. However, these risks remained significantly higher than those in the general population. While it is impossible to completely rule out the delayed diagnosis of very slowly growing brain tumors, the fact that the risk remained significantly higher (double) than the general population even with a 5-year lag, supports the increased likelihood of new brain tumor development in patients with OMCNP without a pre-existing tumor. Thus, in addition to the risk of belatedly discovering a brain tumor originally causing the OMCNP, there is a risk of new brain tumor development in patients who initially have OMCNP without an actual brain tumor.

The association between OMCNP and primary malignant brain tumors might be due to the existence of common causal factors between the two diseases. Representatively, head trauma, brain infections, and inflammation are not only causes of OMCNP, but are also risk factors for brain tumors [10,16,17,18,19,20,21]. The causal relationship between inflammation and cancer is widely accepted [22,23,24,25]. Chronic inflammation may induce DNA damage and genetic instability that can cause malignant transformation through mutation or epigenetic pathways [23,24,25]. Head trauma may also induce malignant brain tumors through cell damage, inflammation, and repair processing [8,26,27]. A previous study has highlighted molecular mechanisms that entities such as p53, hypoxia-inducible factor 1α, and c-MYC play important roles in the relationship between head trauma and glioblastoma [7]. It is conceivable that these elements initially manifest as OMCNP, and pave the way for primary malignant brain tumor development over time. HIV infection is known to increase the risk of not only primary brain lymphoma, but also other types of primary brain tumors [28,29]. However, in the present study, there was no significant difference in HIV infection between the two groups. In addition, the effect of HIV infection on primary malignant brain tumors could not be proven due to a low prevalence of HIV in South Korea [30,31].

Our subgroup analysis showed that younger women (below 65 years) diagnosed with OMCNP were especially vulnerable to primary malignant brain tumors. This finding is likely attributable to OMCNP’s etiological variation across age groups. While the elderly predominantly exhibit microvascular ischemia as the primary cause of OMCNP, younger individuals lean towards trauma, inflammation, and infections [17,18]. Therefore, age-specific OMCNP etiologies might elucidate the differential brain tumor risks seen in these groups.

A limitation of this study was its retrospective design, which limited the determination of cause and effect between OMCNP and primary malignant brain tumors. Our data could only suggest a correlation between the two diseases without predicting a causative relationship between them. Secondly, we could not analyze histopathologic details of tumors, and whether recovery from OMCNP was associated with the risk of primary malignant brain tumors due to the nature of the KNHIS. Further large prospective cohort studies are needed to confirm that OMCNP is a causal risk factor for primary malignant brain tumors and to determine whether the recovery from OMCNP affects the risk of primary malignant brain tumors. Nonetheless, our research holds significance by spotlighting the heightened primary malignant brain tumor risk among OMCNP patients by leveraging a comprehensive nationwide cohort.

## 5. Conclusions

OMCNP is a salient predictor for subsequent primary malignant brain tumors, with women diagnosed before age 65 being especially susceptible. Therefore, consistent surveillance for primary malignant brain tumors in OMCNP patients aged below 65 years is advocated even if brain tumors are not detected on neuroimaging during the initial evaluation.

## Figures and Tables

**Table 1:** Demographics of participants with cranial nerve palsies and controls.

	Total *N* = 118,686	Control Cohort *N* = 98,905	OMCNP Cohort *N* = 19,781	*p*-Value
Sex, men, *N* (%)	74,778 (63.0)	62,315 (63.0)	12,463 (63.0)	1.0000
Age, years	59.82 ± 13.05	59.82 ± 13.05	59.82 ± 13.05	
≥65 years, *N* (%)	47,394 (39.93)	39,495 (39.93)	7899 (39.93)	1.0000
Smoking, *N* (%)	26,318 (22.17)	22,014 (22.26)	4304 (21.76)	0.1227
Drinking, *N* (%)	51,462 (43.36)	43,451 (43.93)	8011 (40.50)	<0.0001
Regular physical activity, *N* (%)	25,379 (21.38)	21,104 (21.34)	4275 (21.61)	0.3909
Low income, *N* (%)	23,973 (20.20)	20,126 (20.35)	3847 (19.45)	0.0040
Obesity, *N* (%)	44,288 (37.32)	36,386 (36.79)	7902 (39.95)	<0.0001
Diabetes mellitus, *N* (%)	21,978 (18.52)	16,029 (16.21)	5949 (30.07)	<0.0001
Hypertension, *N* (%)	53,620 (45.18)	43,508 (43.99)	10,112 (51.12)	<0.0001
Dyslipidemia, *N* (%)	39,819 (33.55)	31,729 (32.08)	8090 (40.90)	<0.0001
Chronic kidney disease, *N* (%)	8970 (7.56)	7350 (7.43)	1620 (8.19)	0.0002
HIV infection, *N* (%)	8 (0.01)	5 (0.01)	3 (0.02)	0.1138

OMCNP, ocular motor cranial nerve palsies; *N*, numbers; HIV, human immunodeficiency virus.

**Table 2:** Hazard ratio for primary malignant brain tumors.

	Number	Event	Person-Years (PYs)	Incidence Rate (per 1000 PYs)	HR (95% CI)		
					Model 1	Model 2	Model 3
Control cohort	98,905	81	437,474.19	0.185	1 (reference)	1 (reference)	1 (reference)
OMCNP cohort	19,781	52	86,700.57	0.600	3.239 (2.287, 4.589)	3.241 (2.288, 4.591)	3.272 (2.294, 4.665)

PYs, person-years; HR, hazard ratio; CI, confidential interval; OMCNP, ocular motor cranial nerve palsies. Model 1: unadjusted. Model 2: adjusted for age and sex. Model 3: adjusted for age, sex, low-income, smoking, drinking, regular physical exercise, hypertension, diabetes, dyslipidemia, obesity, chronic kidney disease, and human immunodeficiency virus infection.

**Table 3:** Hazard ratio for primary malignant brain tumors with 3-year or 5-year time lag.

		Number	Event	Person-Years (PYs)	Incidence Rate (per 1000 PYs)	HR (95% CI)		
						Model 1	Model 2	Model 3
3-year time lag	Control cohort	79,889	44	245,254.96	0.179	1 (reference)	1 (reference)	1 (reference)
	OMCNP cohort	15,853	21	48,276.43	0.435	2.425 (1.442, 4.078)	2.425 (1.442, 4.077)	2.39 (1.406, 4.061)
5-year time lag	Control cohort	50,482	20	112,716.58	0.177	1 (reference)	1 (reference)	1 (reference)
	OMCNP cohort	9925	8	22,090.85	0.362	2.041 (0.899, 4.634)	2.042 (0.900, 4.637)	2.055 (0.892, 4.735)

PYs, person-years; HR, hazard ratio; CI, confidential interval; OMCNP, ocular motor cranial nerve palsies. Model 1: unadjusted; Model 2: adjusted for age and sex; Model 3: adjusted for age, sex, low-income, smoking, drinking, regular physical exercise, hypertension, diabetes, dyslipidemia, obesity, chronic kidney disease, and human immunodeficiency virus infection.

**Table 4:** Locations of primary malignant brain tumors in participants with cranial nerve palsies and controls.

Locations	Total	Control Cohort	OMCNP Cohort	
Event/Number	133/118,686	81/98,905	52/19,781	*p*-Value
				0.0047
Meninges	16 (12%)	4 (5.0%)	12 (23.1%)	
Brain parenchyma	104 (78.2%)	70 (86.4%)	34 (65.4%)	
Other central nerve system	13 (9.8%)	7 (8.6%)	6 (11.5%)	

**Table 5:** Subgroup analysis for other factors on association of ocular motor cranial nerve palsies with primary malignant brain tumors.

Subgroups		HR (95% CI)	*p* for Interaction
Sex	Men	2.441 (1.532, 3.889)	0.0440
	Women	5.118 (2.933, 8.932)	
Age	<65 years	6.951 (3.995, 12.094)	0.0006
	≥65 years	1.899 (1.158, 3.114)	
Smoking	No	3.420 (2.308, 5.067)	0.6162
	Yes	2.724 (1.219, 6.086)	
Drinking	No	3.150 (2.022, 4.905)	0.7765
	Yes	3.497 (1.961, 6.236)	
Regular physical activity	No	3.049 (2.030, 4.580)	0.4769
	Yes	4.096 (2.012, 8.340)	
Low income	No	2.924 (1.949, 4.387)	0.2446
	Yes	4.762 (2.316, 9.793)	
Obesity	No	3.664 (2.372, 5.661)	0.3907
	Yes	2.657 (1.459, 4.837)	
Diabetes	No	3.531 (2.334, 5.342)	0.4985
	Yes	2.696 (1.388, 5.235)	
Hypertension	No	3.646 (2.247, 5.917)	0.5257
	Yes	2.909 (1.746, 4.846)	
Dyslipidemia	No	3.656 (2.423, 5.518)	0.3173
	Yes	2.450 (1.249, 4.807)	
Chronic kidney disease	No	3.258 (2.247, 4.724)	0.9401
	Yes	3.412 (1.080, 10.783)	

HR, hazard ratio; CI, confidential interval.

## Data Availability

Data that support the findings of this study are available from Health Insurance Review and Assessment (HIRA) of Republic of Korea. However, HIRA’s audited approval is mandatory to have access to these data. Thus, these data are not publicly available. However, they are available from the corresponding author upon reasonable request.
